# Identifying niche‐mediated regulatory factors of stem cell phenotypic state: a systems biology approach

**DOI:** 10.1002/1873-3468.12559

**Published:** 2017-01-30

**Authors:** Srikanth Ravichandran, Antonio del Sol

**Affiliations:** ^1^ Luxembourg Centre for Systems Biomedicine (LCSB)University of LuxembourgLuxembourg

**Keywords:** niche determinants, stem cell niche, systems biology

## Abstract

Understanding how the cellular niche controls the stem cell phenotype is often hampered due to the complexity of variegated niche composition, its dynamics, and nonlinear stem cell–niche interactions. Here, we propose a systems biology view that considers stem cell–niche interactions as a many‐body problem amenable to simplification by the concept of mean field approximation. This enables approximation of the niche effect on stem cells as a constant field that induces sustained activation/inhibition of specific stem cell signaling pathways in all stem cells within heterogeneous populations exhibiting the same phenotype (niche determinants). This view offers a new basis for the development of single cell‐based computational approaches for identifying niche determinants, which has potential applications in regenerative medicine and tissue engineering.

## Abbreviations

**MS**, multiple sclerosis

**PCST**, Prize Collecting Steiner Tree; NSC, neural stem cell

Stem cells are indispensable for maintaining tissue homeostasis due to their unique ability to generate more specialized cell types in a well‐coordinated manner depending on the organismal needs. This function depends crucially on the ability of stem cells to make robust cell fate choices such as self‐renewal or differentiation. Multiple cell‐intrinsic and extrinsic factors control this decision‐making process. In this regard, interactions between stem cells and their microenvironment, also known as the niche, determine the stem cell phenotypic states such as quiescent and active stem cells 1. The cellular niche translates information from the neighborhood of the stem cell by transmitting external cues to intracellular signaling events that maintains its cellular state. Schofield in his description of hematopoiesis, proposed the concept of stem cell niche where, a stem cell must be associated ‘with other cells which determine its behavior’ in order to ‘prevent its maturation’; loss of this association was hypothesized to result in differentiation 2. This concept of stem cell niche has evolved over time, and now includes several different supportive stromal cell types, anatomical localization, soluble molecules, as well as physical factors, such as shear stress, oxygen tension, and temperature 3. Involvement of such disparate and stochastically fluctuating components, in addition to feedback regulation of the niche by stem cells, leads to the highly dynamic nature of the niche 1, 4, 5. Stem cells are known to remodel the niche by secreting ECM components and other diffusible factors in response to the signals received from the niche, thus giving rise to feedback regulation of niche–stem cell interactions 4. Such a bidirectional interplay between stem cells and niche is exemplified by the fact that daughter/progenitor cells can serve as niche cells for their parent stem cells in different tissue types 1. These feedback regulatory mechanisms, in addition to the complex bio‐physical characteristics of ECM, contribute to nonlinear stem cell–niche interactions 6.

General physiological conditions of tissue and organismal requirements shape the niche effect on stem cell phenotype 7. For instance, healthy tissues under homeostatic conditions are characterized by the tight regulation of stem cell and progenitor cell turnover. However, this tissue‐level homeostasis is often disrupted in case of several diseases such as cancers, neurodegenerative diseases, and cardiac dysfunction. Furthermore, aging is known to contribute toward progressive decline in tissue homeostasis due to degenerative changes in niche‐mediated cues that regulate the stem cell activity 8. In general, complications often arise due to a lack of proper generation of progenitor cells, complete loss of stem cells, and uncontrolled growth of stem/progenitor cells. Deregulated niche components are known to be responsible for several of these defects 9. For such cases, regenerative medicine approaches that rely on transplanting or modulating endogenous stem cells hold immense potential 9. At present, a key challenge in this area includes the limited functional integration (or engraftment) of transplanted stem cells into the target tissue. This has been attributed to the negative regulatory effect of diseased niche on transplanted stem cells 10. In order to overcome this limitation, it is essential to understand those regulatory mechanisms that normally control stem cell functional state in response to the niche. However, the multifactorial complexity of the niche–stem cell interactions is a major roadblock in this direction. Therefore, the role of the niche in maintaining distinct stem cell phenotypic states, and how to influence the niche effect on stem cells to induce transitions among these states constitutes a fundamental problem in stem cell research.

Recently, studies have begun to address this issue by explicit characterization of niche components and their interactions with stem cells 11, 12. Despite significant progress in identifying cells that comprise the niche, a comprehensive understanding of all niche components is not yet obtained. This lack of knowledge is predominantly due to the difficulty in obtaining and studying niche cells and factors *in vivo*. Furthermore, there is a lack of consensus on what actually constitutes the niche and the precise definition of niche components 13, 14, 15. In addition to experimental efforts, a few computational systems biology approaches that model population‐level dynamics of cell–cell interactions have been proposed to study niche regulation of stem cells 16, 17, 18, 19, 20. However, a complete description of stem cell–niche interactions that allows designing strategies for controlling the effect of niche on stem cells is still limited. This is mainly due to incomplete characterization of the niche, fluctuations of the niche components, and a large number of nonlinear interactions between the niche components and stem cells.

In this article, we hypothesize that stem cell–niche interactions could be considered as a complex many‐body problem that can be simplified by the concept of mean field approximation. Such a view allows consideration of the net effect of all niche components on stem cells as a constant averaged effect or ‘mean field’. Most existing models consider the niche composition to model stem cell–niche interactions via rate equations. Our approach does not require this knowledge, precisely because it considers that stem cells interact with their niches via a mean field created by all niche components, which ultimately determines the sustained activation/inhibition of specific stem cell‐signaling pathways that maintain their phenotypic states. Application of our view allows the identification of niche‐mediated regulators of stem cell phenotypes by relying on single‐cell profiling data. To support this hypothesis, we use examples of different stem cell systems to illustrate how stem cells maintain their phenotypic state via constant activation or inhibition of certain pathways under homeostatic conditions. Such pathways that determine the stem cell states can be termed as niche determinants, and are expected to be constantly activated/inhibited in all cells within a population sharing the same phenotypic state despite the variability in their molecular profiles. Indeed, knowledge of these niche determinants should enable us to identify target genes whose perturbations can induce transitions between different phenotypic states.

## Mean field approximation: keeping it simple

Mean field theory was initially developed by Pierre Curie and Pierre Weiss in physics for a simplified theory of ferromagnetism 21, 22. They considered a lattice composed of magnetic moments interacting with their nearest neighbors, and proposed to replace the actual interactions experienced by each magnetic moment with the mean interaction (provided by the mean magnetization) by setting the fluctuations around the mean equal to zero. Such an approximation that considers each magnetic moment to be influenced by a mean field created by all their neighboring moments enabled Curie and Weiss to effectively simplify the many‐body interaction problem to a two‐body problem without explicitly accounting for each pairwise interaction. Since its initial proposal, different interpretations of mean field theory have been applied to other disciplines, such as ecology, epidemiology, and protein structure prediction 23, 24, 25.

## Mean field approximation applied to the stem cell niche

**Figure 1:**
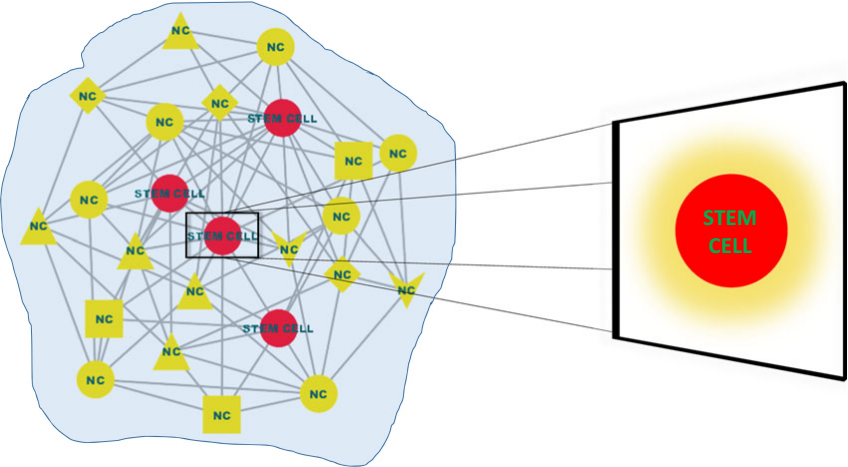
Mean field approximation of stem cell–niche interactions. The mean field approximation considers that each stem cell interacts with its niche via a ‘mean field’ created by all molecular and cellular signals from the niche. The figure depicts the complex nature of stem cell–niche interplay within a spatial compartment. Stem cells (red circles) are entangled in an intricate network of interactions (gray edges) with different niche components (NC) (yellow nodes of different shapes). Analyzing the effect of each individual component on stem cell would require consideration of a large number of interactions and fluctuations among them. In the right, the enlarged depiction of a stem cell shows a mean field (yellow cloud) created by the niche components around a stem cell.

**Figure 2:**
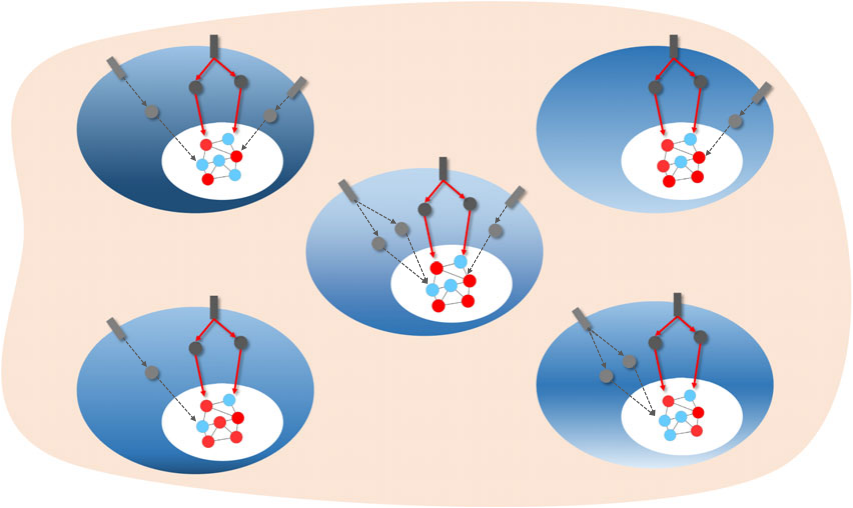
Niche determinants of stem cell phenotype. Representation of stem cell signaling and gene regulatory network states of a heterogeneous population of stem cells sharing a common phenotypic state. The figure depicts heterogeneity of gene expression at a single‐cell level (red and blue nodes) and the signaling pathways regulating the underlying gene regulatory network. According to the mean field hypothesis, in spite of molecular heterogeneity and fluctuations of niche signals, these cells should share commonly activated/inhibited signaling pathways (niche determinants) that determine their phenotypic state. Such pathways are depicted with red arrows, while the other transient signaling pathway activities not common to all cells in the population are depicted with dashed arrows. The underlying gene regulatory network that maintains the phenotype of these cells is depicted with red and blue nodes representing their expression status.

Despite the existence of different mean field concepts 24, here, we follow the definition proposed in ferromagnetism. In particular, we hypothesize that stem cells and niche components within a spatial compartment can be viewed as a many‐body interaction system that includes different types of interactions among them (Fig. 1). By stem cell niche we invoke the original concept of specialized microenvironment which supports stem cell survival and functions 1, 2. In this regard, even though individual components of the niche can fluctuate, their combinatorial effect on stem cells can be represented by a mean field, which is the average of all the molecular and cellular signals from the niche. A single component of the niche may be perturbed, but it does not form a defective field unless the perturbations spread and completely transforms the entire niche 1. The dynamic equilibrium between the niche and the stem cells is resilient and robust to small perturbations and noise in the individual niche components. Therefore, according to our hypothesis, it is not the interaction between stem cells and individual niche components that determines their state, but rather it is the constant interaction of each stem cell with the mean field that leads to a sustained activation or inhibition of specific stem cell intracellular signaling pathways. This ultimately dictates stem cell function and behavior, governing the choice between quiescence, proliferation, self‐renewal, or differentiation. In this way, not only are discrete fluctuations in niche signals buffered against, but so too are the epigenetic and gene expression heterogeneity that stem cell populations display. According to our view, a given stem cell population (sharing a common phenotype), although exposed to perturbations and noise due to fluctuations in individual niche components in addition to the presence of intrinsic molecular heterogeneity, nonetheless should share commonly activated/inhibited signaling pathways that determine their phenotypic state (Fig. 2). Such pathways that determine the stem cell state can be termed as niche determinants (Fig. 2).

As a consequence of approximating the niche components with an effective mean field, the focus is on identifying sustained signaling (shared within a cellular population) responsible for maintaining the specific stem cell phenotype instead of characterizing the niche explicitly. The proposed approach relies on single‐cell profiling data and works by first identifying the most conserved set of genes (based on the similarity of expression levels at single‐cell resolution) defining that particular phenotype. Subsequently, unique signaling pathways/networks that link the conserved receptors and transcription factors for specific stem cell phenotypes are inferred computationally by relying on network topology and expression levels.

## Case study: mean field approximation to identify niche determinants of NSCs

Based on a mean field approximation hypothesis, we illustrate the applicability of this view of stem cell–niche interactions in order to identify niche determinants of quiescent and active neural stem cell (NSC) phenotypes based on a recently published single‐cell RNA sequencing data 26. The data were obtained from Gene Expression Omnibus (GSE67833). Briefly, these data that we used in our approach are described as follows: mouse subventricular zone NSCs were isolated from their natural environment based on the expression of GLAST and Prom1. The transcriptome of 104 GLAST+/Prom1+ cells were analyzed by single‐cell RNA‐seq using Smartseq2 technology 27. These data were then subjected to principal component analysis followed by unsupervised hierarchical clustering of genes with the highest coordinates in the first four principal components (1844 genes) 26. This analysis partitioned the NSCs into two major clusters. One NSC cluster had *Egfr* expression (a known marker of active NSCs 28) in addition to the expression of cell cycle‐related genes. Based on these attributes, this cluster was defined as active NSCs. On the other hand the cluster that lacked the activation markers were classified as quiescent NSCs. Gene ontology and pathway enrichment analysis revealed that active NSCs were enriched in genes for cell cycle, protein synthesis, and mitosis, whereas glycolytic metabolism was found to be most enriched in quiescent NSCs. Gene ontology and pathway enrichment analysis further divided quiescent and active NSCs into two subpopulations each (quiescent NSC1/2 and active NSC1/2). In our current analysis for the sake of simplicity we considered only quiescent and active NSC populations as a whole without considering the further subpopulations.

Our strategy relies on gene expression differences between stem cells displaying different niche‐dependent phenotypes, and aims to infer sustained signaling pathways that are required for stably maintaining their corresponding phenotypes. Moreover, despite the niche‐induced fluctuations in signaling, such pathways must be shared (or conserved) within the cells sharing a common phenotype. However, it must be mentioned that identification of conserved pathways can also result in housekeeping pathways that could be of general importance to a wide variety of cell populations (e.g., pathways that are important for both quiescent and active NSCs) and therefore could lack cell type specificity. In order to overcome this issue, the approach focuses on uniquely conserved pathways within each population and is different across the populations.

Single‐cell gene expression data offer the possibility to identify the set of genes whose expression pattern is conserved within a given phenotype. Such genes are more likely to play a dominant role in phenotype maintenance since their expression pattern is similar at single‐cell level. In the example of NSCs, we first identified the genes exhibiting similar expression pattern within quiescent or active phenotype. For this we employed Shannon entropy 29, which measures the disorder of a system, where lower values indicate similar expression pattern of a given gene. Entropy for each gene, *X*, is defined by:

$$H\left(X\right)=-\sum_{i\;=\;1}^{n}P\left(x_{i}\right)\log_{2}p\left(x_{i}\right)$$

where *P(x*_*i*_ *)* represents probability of gene expression value *x = x*_*i*_. Entropy calculation was performed using data binning approach and the number of bins (*k*) was determined from the expression data using Sturges' rule 30, given by *k* = log_2_ *n* + 1, where *n* is the sample size. After data binning, the computation of entropy was performed using maximum likelihood implementation (entropy.empirical) of the R entropy package. We used an entropy cutoff less than 1 and median expression (FPKM) value greater than 10 to classify the gene as having a conserved expression pattern. Entropy calculation for each gene allowed us to identify quiescent or active phenotype‐specific genes that showed similar expression pattern at a single‐cell level.

Next, we sought to identify those signaling pathways that are more likely to be constantly active. For this, we first identified the set of receptors/ligands and transcription factors classified as conserved for quiescent and active NSCs. Entropy calculation based on single‐cell expression levels allowed us to identify the genes that shared a similar expression levels. From that list of genes, transcription factors and transcriptional regulators were identified based on annotation available at Animal TFDB (http://www.bioguo.org/AnimalTFDB/). In the case of receptors, since a complete database of receptor molecules is currently unavailable, we used Gene Ontology classification of receptor activity and plasma membrane (GO:0004872, GO:0005886) to identify genes with possible receptor activity. For the case of secreted ligand molecules we utilized the classification of potential ligands reported in a recent study 31. About 90 and 128 receptors/ligands were identified for quiescent and active NSC phenotypes, respectively. From this, identifying the ones that are most likely to propagate the niche mediated signaling is a challenge. We made use of literature‐curated signaling database Reactome 32 as a background raw signaling network consisting of all reported signaling interactions and employed Prize Collecting Steiner Tree (PCST) formalism to infer the signaling pathways. Interactions reported in the Reactome database were used as the background network from where subsequent Steiner trees were inferred. Reactome consists of curated pathways with molecular interaction data from Reactome Functional Interaction Network and other databases such as IntAct, BioGRID, ChEMBL, iRefIndex, MINT, and STRING. We specifically used Reactome Functional Interaction Network (http://www.reactome.org/pages/download-data/) as they contain information on direction and sign (positive of negative regulatory effect) of the interaction. We consider that the conserved receptors/ligands of a given stem cell phenotype are under the direct influence of the niche. Since the exact mechanisms of the niche effect on the signaling activity are not known, we represent the net effect of the niche by introducing a dummy niche node in the raw signaling network. The external dummy node is incorporated as a way to capture the topologically favorable receptors/ligands (from several expressed ones) that can link it to the TFs specific for quiescent and active NSCs. Furthermore, the dummy node is used as the root node which acts as the starting point for Steiner tree identification, consequently the receptors/ligands will be linked to the dummy node in the inferred Steiner trees. This dummy node is connected to all conserved receptors/ligands for each phenotype under consideration. Therefore, signal transduction from the niche to transcription factor must be propagated through at least one of the conserved receptors. The edges in the signaling interactome were weighted using the gene expression data, where the weights were calculated as, $c_{e}=\frac{1}{x_{i}x_{j}}$, where *x*_*i*_ and *x*_*j*_ are the expression levels of the interacting nodes. We specifically used such a weighting scheme since the objective of the PCST algorithm is to collect as many high prize nodes (genes with high expression) while minimizing the edge weights. Such an edge weighting scheme that inversely correlates with the expression levels will enable collecting those edges where both nodes are highly expressed. In such a weighted raw signaling network, that has a dummy niche node representing the net effect of the niche, we used PCST to infer subnetworks with the dummy niche node as the root or origin node and the conserved transcription factors as the terminal nodes. Steiner Tree formalism has been used earlier to reconstruct active signaling pathways 33, 34. Formally, the PCST problem is defined as, given a graph *G* = *(V,E)*, representing the raw signaling interactome (where, *V* denotes the nodes and *E* denotes the edges), with defined edge costs (weights), *c*_e_ and node prizes *b*_*v*_ find a connected subgraph *T = (V′,E′), V′* ⊆ *V, E′* ⊆ *E,* that minimizes the following function:

$$T=\underset{(E^{\prime },V^{\prime })\mathrm{connected}}{\text{min}}\left(\sum_{e\in E^{\prime }}c_{e}-\lambda \sum_{v\in V^{\prime }}b_{v}\right)$$

The node prizes are computed by *b*_*v*_= |log fold change (*V*)| from the gene expression data and *c*_e_ is the edge weights. The constant λ determines the tradeoff of adding new proteins to the inferred network by balancing the cost of new edges and the prize gained by adding a new protein. We chose λ = 0.01 for our simulations and employed a heuristic method based on a message‐passing algorithm to infer the PCSTs 33. Basically, minimizing this function implies collecting the largest set of high prize nodes while minimizing the set of high cost edges in a tradeoff tuned by λ that results in a connected subgraph. Since the dummy node is connected only to the conserved receptors of a given cell type, the inferred subnetworks will encompass only those receptors that are both topologically favorable and maximize the expression values of the intermediate nodes. Therefore, from several conserved receptors, one could narrow down to the few linking the transcription factors based on their unique network topological features and expression levels.

Employing the above strategy, we identified subnetworks that are likely to maintain the quiescent and active phenotypes of NSCs (Fig. S1). In the case of quiescent NSCs, we identified nine subnetworks with receptors as origins/sources responsible for controlling the expression status of the downstream terminal transcription factors (Fig. S2). Among such identified receptors, the role of Bmpr1b, Notch2, and S1pr1 are known in the case of quiescent NSCs. In fact, BMP signaling is known to maintain the NSC quiescence in an autocrine manner, and further this signaling must be downregulated for the subsequent activation of the quiescent NSCs 26. On the other hand, Notch signaling is known to be involved in a paracrine manner where Notch ligands are expressed by active NSCs and inhibition of Notch signaling increased the active stem cell population 26. Role of S1pr1 in maintaining NSC quiescence has been demonstrated in an independent study where addition of S1pr1 agonist sphingosine‐1‐phosphate significantly affected the activation of quiescent NSCs 28. In the case of active NSCs, we identified Egfr signaling in addition to five other receptor‐mediated signaling pathways(Fig. S3). Moreover, role of Egfr signaling for maintaining active NSCs is well established and in fact Egfr is used as a marker to isolate those cells 28. In principle, such an approach that focuses on sustained signaling pathways conserved within a cellular population could enable identification of niche‐mediated regulators of stem cell phenotypes without the knowledge of niche.

## Mean field approximation: caveats and comparisons to other models

As a result of mean field approximation, transient fluctuations in signaling events that arise due to the dynamic nature of the niche are ignored, as they do not display any functional consequence for the maintenance of stem cell states. In this context, it must be noted that in addition to sustained signals, a cellular niche can also propagate transient, but functionally relevant signals induced by feedback mechanisms to robustly maintain tissue homeostasis 35. Other transient, yet functionally important signals could arise due to perturbations such as cellular injury or genomic mutations. The latter signals generally induce stem cell phenotypic transitions (i.e., from quiescent to active/proliferative state 5), but are less likely to stably maintain the existing stem cell phenotype 36, 37. Therefore, it must be emphasized here that the mean field view of stem cell–niche interactions is valid for identifying the signaling pathways responsible for constant maintenance of cellular phenotypes and not for transient signals that can potentially trigger phenotypic transitions. Furthermore, identification of conserved signaling can provide accurate descriptions of individual cellular behavior only when heterogeneity within a defined population reflects functionally meaningless fluctuations around a single cellular state and not otherwise. Therefore, for the approach to yield accurate results, the characterization of the cellular populations needs to be accurate.

Greater emphasis on the identification of sustained signaling pathways that are conserved within a cellular population exhibiting a common phenotype is a major outcome of the mean field approximation of the niche. Even though this outcome appears similar to pathway enrichment analysis that has been routinely utilized over the past decade 38 to identify deregulated (signaling or metabolic) pathways, in actual practice the idea has not been identification of sustained signaling pathways conserved within a cellular population. Moreover, several transient signaling pathways could be identified as deregulated due to indirect effects (of mutations, differences in the niche composition etc.) and not as a cause for observed phenotypic difference. However, those signaling pathways that are constantly active are more likely to be the cause for stable maintenance of a specific cellular phenotype. Such a view offered by our hypothesis is fundamentally different from the prevailing view, and is often overlooked due to its apparent simplicity. Furthermore, it must be mentioned that computational analysis based on such a view enhances the utility of single‐cell omics data generation and adds value to current development of analytical methods 39 to decipher hidden patterns in such high‐resolution datasets.

Given the complexity involved in stem cell–niche interactions, computational systems biology approaches have been useful in modeling their behavior. In fact, computational methods have been proposed to model interactions between stem cells and niche components 16, 17, 18, 19, 20, 40, 41, 42. These methods could be broadly classified into two major categories, (a) methods that aim to capture the population level behavior of stem cell–niche interactions by modeling cell–cell interaction dynamics and (b) construction of intercellular (cell–cell) interaction networks based on gene expression data.

The first category of methods model the interaction dynamics of stem and progenitor cells using rate equations that describe the birth and death processes of each cell type and their interdependence on each other 16, 17, 18, 19, 20, 40. Such models are most commonly employed for studying stem cell–niche interaction dynamics and characterizing the system steady‐state properties in order to understand tissue homeostasis, and how perturbations (in the form of diseases) could affect the original steady states. A typical bottleneck in such dynamical models is the lack of knowledge of parameters or probabilities (such as, self‐renewal rate, synthesis rate of differentiated cells, death rates of stem and daughter cells) that govern the system dynamics. In addition to a lack of knowledge on parameters, even the precise composition of the cellular niche is far from being completely known, thereby rendering the development of such dynamical models difficult. Furthermore, these models tend to be powerful for a descriptive analysis of the system dynamics rather than being predictive in nature. In contrast, our proposed approach does not require the explicit knowledge of niche components or the parameters that govern the stem cell–niche interactions to identify niche‐mediated regulators of stem cell phenotype.

The second category of models are based on construction of intercellular interaction networks based on gene expression data 41, 42. This approach attempts to build cell–cell interaction networks based on sorting of different cell populations followed by high‐throughput profiling, to define intercellular signaling between phenotypically defined populations of stem, progenitor, and mature cell types. This approach, although not affected by a lack of knowledge on parameters, nevertheless requires sorting and profiling of several cell types to construct the cell–cell interaction network. This is a major limitation since the cell types that truly serve as niche cells in several stem cell systems is not well characterized, and therefore cannot be sorted and profiled easily. However, our proposed strategy requires single‐cell gene expression profiling of only the stem cells with distinct phenotypes (like quiescent and active) and does not require expression profiling of the niche cells. This dramatically simplifies the isolation of the cells, data generation and further downstream analysis since only stem cells are required to be isolated and profiled without the necessity of profiling the niche cells.

Although every stem cell system is unique in the way it is regulated by its niche 3, several recent studies in different stem cell systems have observed that stem cell states are determined by constant activation/inhibition of specific pathways by the constitutive influence of its niche 15, 28, 43. The presence of certain constantly activated/inhibited signaling pathways maintained by their niche appears to be the commonality in different stem cell systems. This offers possibilities to address the complexity of stem cell–niche interactions without the explicit niche characterization. Especially, the rapid advancements in single‐cell profiling technologies enable the dissection of cellular populations in greater detail. Moreover, the development of computational systems biology approaches based on the mean field approximation hypothesis finds a natural application of such increasingly available data for identifying signaling pathways that are constantly active in all cells within a population exhibiting the same phenotype. Importantly, identification of such niche determinants has several implications in regenerative medicine.

## Potential applications for regenerative medicine and tissue engineering

The stem cell niche contains a rich and diverse set of cues that impinge constantly on stem cells that can be modulated for therapeutic gain 9, 10. Understanding and characterizing the niche determinants has potential applications in regenerative medicine and stem cell therapies for degenerative diseases of liver, heart, lung, and brain. Limited functional integration of transplanted stem cells into the target tissue possibly due to negative regulatory effect of diseased niche is currently a major challenge 10. In this regard, promoting regeneration by harnessing the latent regenerative potential of endogenous stem/progenitor cells has been used as an alternative regenerative medicine strategy in order to overcome the current translational bottlenecks associated with cell transplantation 44. For example, in the case of multiple sclerosis (MS), a demyelinating disease due to progressive failure of remyelination in the CNS due to aging, endogenous activation of oligodendrocyte precursors by mimicking a youthful microenvironment have been proven useful to promote remyelination in certain MS disease models 45, 46. In order to achieve this, identification of strategies for the activation of endogenous repair mechanisms to promote tissue regeneration in situations in which it does not occur normally is necessary 44. Within this context, the proposed approach for the identification of conserved signaling pathways under diseased and healthy niche conditions (determined by their physiological cues) can enable the development of potential strategies to modulate endogenous stem cell activity by either counteracting the effect of diseased niche or by mimicking the effect of healthy niche in the diseased counterpart. Such intervention strategies would be intended to make endogenous stem cells resistant to the perturbed signals in the diseased state and to sustain long‐term function.

Another potential application where the knowledge of niche determinants can provide useful insights is in the area of tissue engineering. In particular, it is relevant in the context of *ex vivo* tissue engineering, where the main goal is to have the cells surviving and functioning in an optimal environment without necessarily having to replicate the *in vivo* conditions. In this regard, our proposed approach can enable identification of key factors that are responsible for maintaining a given cellular phenotype *in vivo* can aid defining better culture conditions for long‐term phenotype maintenance. For instance, long‐term maintenance of primary hepatocytes in a defined culture medium is still a challenge 47. Specifically, identification of a culture system that can facilitate long‐term maintenance of hepatocytes is advantageous for clinical applications such as drug screening and toxicity tests.

## Conclusions

In general, cellular populations with the same functional phenotype exhibit a certain degree of heterogeneity in their molecular profiles due to intrinsic stochasticity in the transcriptional and translational program. Furthermore, the dynamical nature of the niche can perpetuate noisy fluctuations in stem cell signaling pathway activities. Therefore, stem cells face an acute challenge of robustly maintaining their state in the presence of intracellular and extracellular fluctuations, while responding precisely to developmental cues from the niche. The existence of a common stem cell phenotype within a spatial compartment of a tissue, despite the dynamic nature of the niche, seems contradictory. Our mean field view of stem cell–niche interactions provides an explanation for such a seemingly contradictory observation. By focusing on the net effect of the niche created by the mean field after disregarding internal and external fluctuations, it points to the existence of constantly activated/inhibited signaling pathways that maintains the stem cell state in response to the niche. In fact, identification of conserved signaling pathways that are constantly activated/inhibited in all cells in a stem cell population exhibiting the same phenotype will confirm our hypothesis. Furthermore, the development of single‐cell data‐based computational methods relying on a mean field view of the niche can aid in identification of niche determinants by simplifying the complexity of stem cell–niche interactions. Importantly, the knowledge of niche determinants will aid developing regenerative medicine strategies to enhance/modulate stem cell activity for the treatment of injury, disease, or age‐related dysfunctions. In addition, our approach is suitable for identifying factors that can facilitate long‐term maintenance of cells under culture conditions. Thus, combining recent developments in single‐cell technologies and stem cell research with the systems biology approaches discussed here should enable us to more accurately identify niche determinants, which in turn could lead to the implementation of more feasible strategies in regenerative medicine and tissue engineering.

## Author contributions

AdS conceived the idea. SR performed the analysis. Both the authors wrote the manuscript.

## Supporting information


**Fig. S1.** Inferred Steiner trees for quiescent and active NSCs. It can be seen that the dummy node in the center is the root node that connects with all receptors/ligands. The inverted triangles depict receptor molecules, circles depict signaling intermediates, and squares depict transcription factors.

**Fig. S2.** The figure shows the subnetworks of signaling pathways identified for quiescent NSCs. The inverted triangles depict receptor molecules, circles depict signaling intermediates, and squares depict transcription factors. The experimentally validated signaling pathways are highlighted.

**Fig. S3.** The figure shows the subnetworks of signaling pathways identified for active NSCs. The inverted triangles depict receptor molecules, circles depict signaling intermediates, and squares depict transcription factors. The experimentally validated signaling pathways are highlighted.
