# Supplementary material for: Identifying niche‐mediated regulatory factors of stem cell phenotypic state: a systems biology approach
Source: FEBS Lett. 2017 Jan 30;591(3):560–9. doi: 10.1002/1873-3468.12559 (PMC5324585; doi:10.1002/1873-3468.12559)
Supplement: Supplementary file 1 — Fig. S1. Inferred Steiner trees for quiescent and active NSCs. It can be seen that the dummy node in the center is the root node that connects with all receptors/ligands. The inverted triangles depict receptor molecules, circles depict signaling intermediates, and squares depict transcription factors. [file FEB2-591-560-s001.pdf]

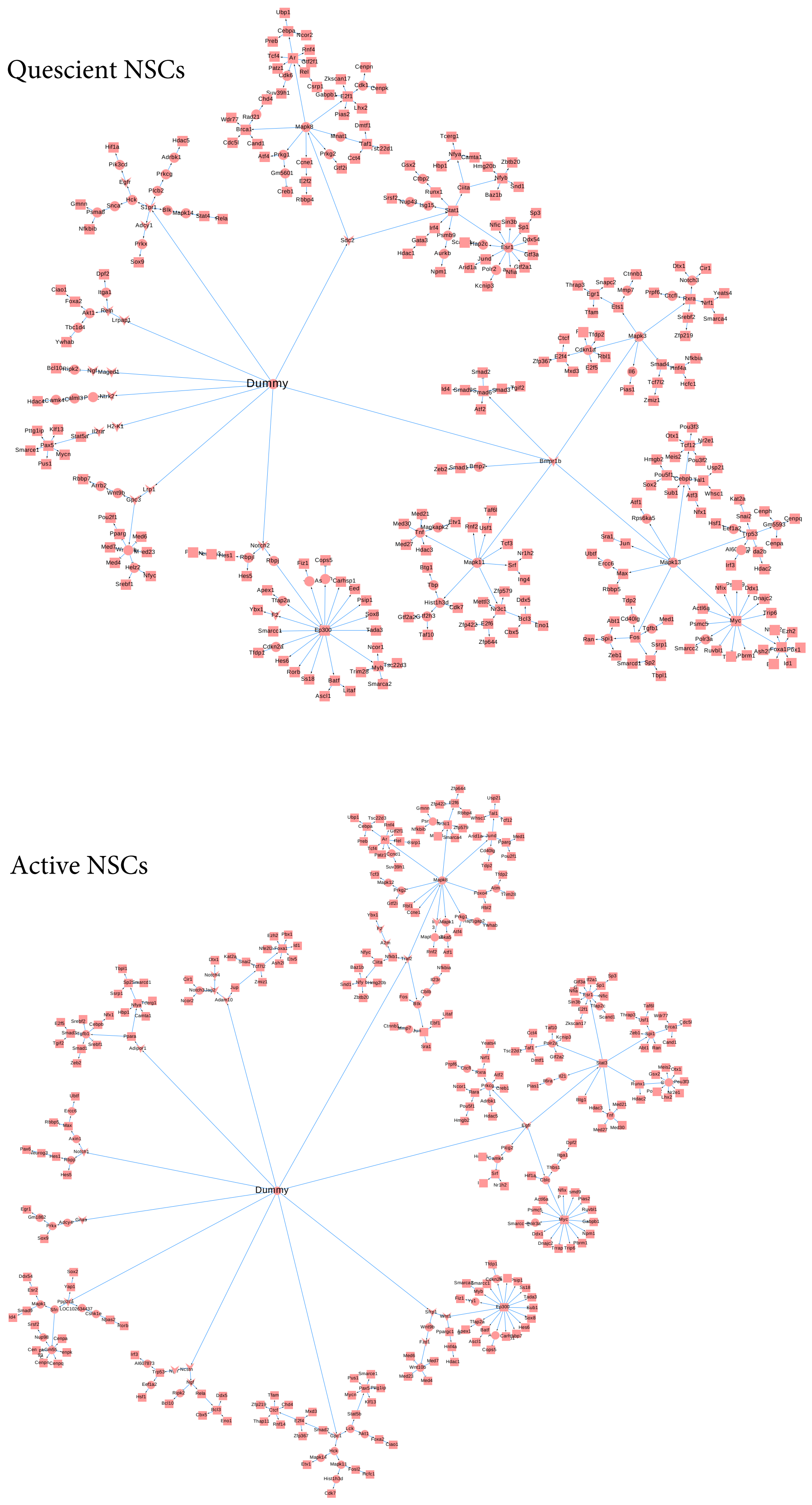

Supplementary Fig. 1

Inferred Steiner trees for quiescent and active NSCs. It can be seen that the dummy node in the center is the root node that connectes with all receptors/ligands. The inverted triangles depict receptor molecules, circles depict signaling intermediates and squares depict transcription factors.
