# Supplementary figures and images for: Identifying niche‐mediated regulatory factors of stem cell phenotypic state: a systems biology approach
Source: FEBS Lett. 2017 Jan 30;591(3):560–9. doi: 10.1002/1873-3468.12559 (PMC5324585; doi:10.1002/1873-3468.12559)

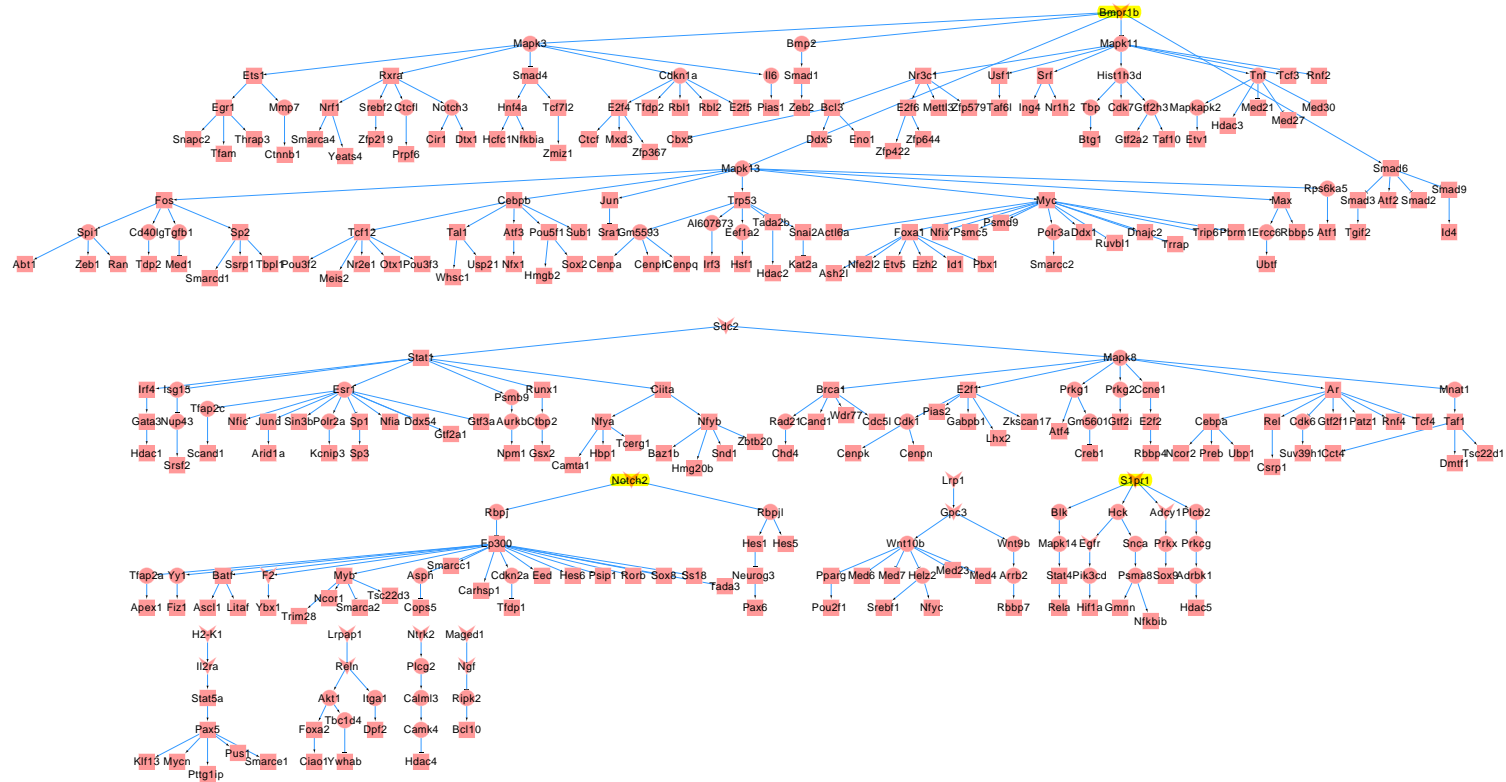

Supplement: Supplementary file 2 — Fig. S2. The figure shows the subnetworks of signaling pathways identified for quiescent NSCs. The inverted triangles depict receptor molecules, circles depict signaling intermediates, and squares depict transcription factors. The experimentally validated signaling pathways are highlighted. [file FEB2-591-560-s002.pdf]
